# Gender inequality in the culinary profession in tourism from the perspective of university students with working experiences in culinary

**DOI:** 10.3389/fsoc.2024.1323096

**Published:** 2024-02-14

**Authors:** Tuba Türkmendağ, Nilgün Karaman

**Affiliations:** ^1^Department of Recreation Management, Faculty of Tourism, Atatürk University, Erzurum, Türkiye; ^2^Department of Gastronomy and Culinary Arts, Faculty of Tourism, Balıkesir University, Balıkesir, Türkiye

**Keywords:** gender, gender inequality, professional culinary, supporting female chefs’ careers, importance of chefs’ leadership qualities in terms of gender equality

## Abstract

Gender inequality is a phenomenon that is particularly prevalent in the culinary profession and professional culinary within the tourism sector, especially affecting female employees. The current study is approached in the context of the patriarchal societal structure and the unique characteristics of gender in the labor market in Turkey. In this regard, this study focuses on the views and opinions regarding gender inequality of undergraduate students in gastronomy and culinary arts programs and, associate degree in vocational cooking programs who have previously worked and/or interned in the professional culinary within the tourism sector in Turkey. For this purpose, a quantitative research method has been employed in the study. In order to reveal detailed insights into participants’ evaluations, open-ended questions have also been included in the survey form. The quantitative findings from the study emphasized the importance of issues such as “the approach of managers,” “working hours,” and “promotion pathways” among the situations that cause gender inequality. The importance of “an equal and fair approach to employees,” and “being kind and considerate to staff” were highlighted as essential qualities for being a good leader/head chef. Important factors in supporting the career of female chefs were identified as “equal pay for equal work,” and “ensuring job safety.” Findings obtained from open-ended questions have revealed that female employees are more exposed to gender inequality in the profession. In this context, issues such as “unequal pay, promotion, and working conditions,” “harassment,” “lack of recognition for female employees in the professional culinary,” and “the effects of patriarchal norms” were highlighted. The study contains important findings that can contribute to increasing the representation of female employees in professional culinary within the tourism sector, improving the productivity and competitiveness of businesses, and addressing gender inequality in society.

## 1 Introduction

Gender inequality is the legal, social, and cultural situation in which sex and/or gender determine different rights and dignity for women and men. This situation manifests itself in women’s and men’s unequal access to or enjoyment of rights and their stereotypical social and cultural roles (Farrell, 2020). Gender inequality may arise because women, unlike men, work in jobs where they are more likely to encounter bad working conditions, inequality of opportunity, violence, stress, and sexual exploitation (United Nations World Tourism Organization and the United Nations Entity for Gender Equality and the Employment of Women, 2011). Due to social roles, gender inequality in the tourism sector arises not only in career but also at the stage of entry into business life. Obstacles may arise, such as the fact that women’s work is not welcomed in society for various reasons, that women have to get permission from their husbands to work, especially in societies with low-income and patriarchal societies, and that women’s work does not comply with religious beliefs (Türkmendağ, 2020).

Especially since the 2000s in Turkey, there have been initiatives to achieve gender equality in the labor market; however, the issue of creating employment in this context remains secondary. Particularly, women’s participation in the workforce is at significantly low levels, and both direct and indirect discrimination based on gender continues in the labor market (Görgün Baran et al., 2016). Therefore, women’s participation in the workforce in the context of gender equality should be evaluated not only in economic terms but also in socio-economic and socio-cultural contexts. Economic barriers include factors such as insufficient job opportunities, working in the informal sector, lower wages compared to male employees, long working hours, and poor working conditions, as well as the inadequacy of childcare/elderly care services. Socio-cultural factors, such as the role of women within the family and the low level of gender diversity in workplaces, exacerbate the effects of economic barriers and weaken women’s motivation to join the workforce (Ustabaş and Gülsoy, 2017).

In the Turkish labor market, women tend to have fewer employment opportunities compared to men, and they are often employed in secondary sectors with lower wages, higher turnover rates, and fewer career opportunities (Sarıtaş Eldem, 2015). According to the data from 2021, the labor force participation rates for individuals aged 15 and above are 32.8% for women and 70.3% for men (Turkish Statistical Insitute, 2022). Among the reasons for this situation, lower levels of education, and work experience are indicated. As the education level of women increases, there is a tendency for higher workforce participation and an improvement in the quality of the jobs they are engaged in (Eyüboğlu et al., 2000; Mitra and Singh, 2006; T. R. Prime Ministry, State Planning Organization, World Bank, 2009; Taşseven et al., 2016; Baz and Aslan, 2021). In 2021, the labor force participation rates based on education level for women were as follows: 12.8% for illiterate women, 25.3% for women with education below high school, 32.5% for high school graduates, and 67.6% for women with higher education degrees (Turkish Statistical Insitute, 2022). On the other hand, factors such as marriage, childbirth, and family responsibilities lead to interruptions in women’s entry and exit from the labor market, career interruptions, and acquiring less work experience compared to men. This situation prompts employers to prefer male labor, considering it more efficient (Sarıtaş Eldem, 2015; Al-Asfour et al., 2017; Giray and Aydın, 2019). Additionally, employers highlighting the high costs associated with employing female employees and avoiding female employment contribute to the reluctance in hiring women (Sarıtaş Eldem, 2015). In Turkey, the significant informal employment of women in the labor market leads to a lack of social security and insufficient representation in the workplace, preventing women from gaining enough empowerment in the labor market (Ayta and Şen, 2023).

Positive developments in Turkey’s labor market, which can be characterized as positive discrimination toward women’s employment, have aimed to promote women’s employment and prevent informality (T. R. Prime Ministry, State Planning Organization, World Bank, 2009). However, the results obtained from the distribution of expressions related to perceptions of women’s work, according to gender, in the 2021 Turkey Family Structure Survey, have emphasized the need to address the issues mentioned in women’s employment from the perspective of gender inequality. Additionally, the report highlights the patriarchal societal structure in which both genders generally consider women responsible for household chores (Turkish Statistical Insitute, 2022). Therefore, it is possible to state that in the Turkish labor market, gender-based stereotypes are accepted by both genders, and this situation has an impact on the labor market.

One of the most significant sectors where gender-based discrimination is observed in the business world is the tourism sector. Women working in the sector are exposed to different practices and discrimination based on their gender in their jobs, salaries, working conditions, promotions, and intra-organizational social relationships. Granting certain rights and responsibilities to women based on their gender rather than their qualifications, backgrounds, education, and experiences is the most explicit indicator of discrimination (Albayrak, 2019). In Turkey, the sectors where women are most employed are the agricultural sector and the service sector (Taşseven et al., 2016; Ustabaş and Gülsoy, 2017). The employment rate of women in the service sector is 61.2% (Karaca and Taşseven, 2022). However, even in the service sector, which is expected to be feminized, the employment rates of women are observed to lag behind those of men (Görgün Baran et al., 2016). In the tourism sector, where 54% of the workforce consists of women, analyses related to women’s participation in professional work life reveal that a significant portion of the tourism workforce is composed of women. However, the representation of women at the professional level in service and office-related jobs is inadequate, and they earn 10–15% less than their male counterparts. Additionally, the tourism sector has almost twice as many female employers as other sectors, and women in tourism are more likely to own businesses compared to other industries. Nevertheless, in family businesses operating in the tourism sector, there is a prevalent situation where women work largely for free and are employed informally (United Nations World Tourism Organization, 2019). Therefore, in jobs related to tourism, it is noteworthy that societal perceptions, values, and attitudes regarding women’s employment are influenced by gender roles and expectations, reflecting the characteristics of the gendered labor market in a patriarchal society, along with the reflections of education levels, wage inequality, career opportunities, and working conditions. Widespread sexist attitudes in society can limit women’s involvement in the tourism sector. Particularly, biases regarding women’s leadership abilities in managerial positions can deepen gender inequality. In this context, the fifth article of the United Nations Sustainable Development Goals, the goal of “achieve gender equality and empower all women and girls” (United Nations, 2015), emphasizes the critical role of tourism in ensuring social and occupational gender equality and empowering women.

The sector in which women participate the most as a workforce in tourism is the food and beverage sector. Women are employed in various roles within the professional culinary in the tourism sector, spanning different levels of culinary positions, as well as in functions such as housekeeping, customer service, and upper management. It is important to recruit, retain, and promote talented women into technical and managerial leadership positions to meet the future skills and productivity needs of the industry (Baum, 2013). It is not possible to achieve sustainability in tourism without realizing decent work, gender equality, and the empowerment of all women. Although the tourism sector has provided opportunities for women’s empowerment and gender equality in recent years, there are still many inequalities (Araújo-Vila et al., 2021). Even though women are the gender most associated with cooking, due to the role that society imposes on women, they lag behind male cooks in professional culinary. The role conflict factor, which includes family and work incompatibility, also makes it difficult for women to take part in professional culinary. The fact that housework and cooking become gender-oriented as “women’s work” leads to the devaluation of emotional labor and social and occupational gender inequality. The magical appeal of men’s professional cooking transforms women’s home cooking into a routine task and leads to biased perceptions of their managerial skills. Thus, with the definition of certain roles in society by gender, the gender-based division of labor in professional culinary becomes natural (Kiester, 2016). Therefore, women in professional culinary may face obstacles in their careers, as well as ridicule, humiliation, insults, and harassment. They may also encounter situations where their abilities are questioned, belittled, not given responsibilities, and lag behind men in terms of issues such as salary and promotion. Due to the patriarchal societal structure and the general situation of the gendered labor market in Turkey, the dominance of a male-centric approach, especially in professional culinary within the tourism sector, gives rise to the perception that female employees are in the minority. The search for a solution to gender inequality, which is seen as the main reason for this situation, provides the basis of this study. The lack of research on gender inequality in the professional culinary in tourism and the lack of representation of women in this field reveals the necessity of this research. In this context, the present study is based on the views and thoughts of undergraduate students in gastronomy and culinary arts programs and vocational cooking programs at the associate degree level who have previously worked and/or interned in a professional culinary regarding gender inequality.

## 2 The concept of gender

Sex, while based on biological and morphological foundations to denote the categories of male and female, also the concept of gender encompasses societal and socio-cultural meanings (Deaux, 1985; Becker et al., 2017). A significant portion of what comes to mind when we refer to men and women is associated with the values created by sociocultural interpretations. Therefore, while gender is determined biologically, the concept of gender refers to the perception of gender that has formed socially (Avşar, 2017). In other words, gender can be defined as “the cultural separation of gender roles; the gender roles assigned to women and men socioculturally” (Akbalık, 2013). According to these definitions, there is a significant difference between the concepts of “sex” and “gender.” While the first one describes the physical differences between individuals, the concept of “gender” refers to the societal and cultural meanings attached to those differences and the roles, duties, and responsibilities assigned to men and women by society (Aytekin, 2014).

The concept of “gender” was first introduced by psychiatrist Robert Stoller in his book “Sex and Gender” in 1968. In the book, Stoller proposed the idea that gender is distinct from biological sex (Demirkır, 2022). Ann Oakley, who incorporated the concept of gender into sociology, focused on the subject of how gender roles are learned and in what way in her work titled Sex, Gender, and Society, which was published in 1972, about gender and personality relationships (Vatandaş, 2007). After the studies of Stoller and Oakley, it was understood that biological existence as gender was not sufficient to express differences. The concept of “gender” began to be used to describe social and cultural differences (Akbalık, 2013).

Robert Connell’s work in 1974 on gender has provided an opportunity for the analysis of gender differences (Wedgwood, 2009). Connell’s *Gender and Power Theory* suggests that gender roles and power dynamics based on gender revolve around three fundamental structures. Firstly, there is gender-based division of labor, which examines gender inequalities in economic activities and work life, including issues such as the distribution of work and wage disparities favoring men. Secondly, gender-based power relations analyze power imbalances in relationships and institutions, encompassing topics such as authority, control, and the misuse of power favoring men. The third structure, emotional attachment, explores how social norms and emotional bonds influence gender roles. This structure addresses societal expectations regarding gender-associated behavioral patterns and roles in emotional relationships (Maharaj, 1995; Wingood and DiClemetine, 2002; Kenny, 2007). This theory helps understand how these three structures proposed for the formation and perpetuation of gender roles influence and shape relationships between women and men.

According to Acker (1992), gender can be understood as the evolving way in which differences between women and men are organized in all aspects of social interaction. This definition suggests that gender encompasses the broad patterns of human behavior, customs, and societal arrangements that are shaped by the distinctions between women and men. These activities and practices always have symbolic significance, and gender is a pervasive symbol of power. According to this approach, gender is not a characteristic of individuals, but a process, although the assignment of individuals to gender categories is certainly a central aspect of this process. Gender as socially constructed differences and beliefs that support inequality between women and men, and as identities, is present in all organizations (Acker, 2006). In this context, the concept of gender equality emerges. Gender equality means that there is no discrimination based on a person’s gender in terms of utilizing opportunities, allocating and utilizing resources, and accessing services (Özel Doğan and Piyal, 2017).

In the context of gender equality/inequality, the phenomenon of gender can be examined in relation to the concept of patriarchy. The formation, development, and impact of patriarchal structures in societies vary depending on geographical, cultural, and temporal differences. Throughout this process, changes occur in the roles of women in the social structure and in power dynamics (Hierro, 1994). Patriarchy refers to a comprehensive system that encompasses male dominance prevailing in every aspect of social life and the dominance established over women (Pierik, 2022). In patriarchy, theorized by sociologist Walby (1991), the focus shifts away from the family and the father figure. Instead, emphasis is placed on the construction of a patriarchal structure focusing on the “patriarchal mode of production, patriarchal relations in waged work, patriarchal relations in the state, male violence, patriarchal relations in sexuality, and patriarchal relations in cultural institutions such as religion, media, and education.”

In the patriarchal system, which is a societal structure and practice system where men exert pressure on women and establish dominance, men control and monitor the productivity of women both within the household and in paid employment. The relationships imposed by this system lead to the marginalization of women in both home and work life. The patriarchal system grants superiority to men in social relationships within society, making men more powerful (Özçatal, 2011). In this context, according to social role theory, gender patterns arise from the different distribution of social roles for men and women both at home and in the workplace. Accordingly, women tend to perform the majority of routine household chores at home and play the primary caregiver role. In the workplace, they are inclined to be employed in people-focused, service professions rather than traditionally male-dominated, object-focused, competitive professions (Hentschel et al., 2019). Women resisting the imposition of patriarchal structures and the central role of men has taken place gradually and in various forms across different societies over time. Women’s rights movements, calls for equality, and efforts for social change have questioned and led to changes in patriarchal structures. However, the impact and outcomes of these struggles have varied depending on societies and time periods. Therefore, this study aims to understand the situations within the patriarchal societal structure in Turkey, that lead to gender inequality for women in the culinary profession within the tourism sector.

The differences between genders have been created by societal norms in which men are typically positioned higher in social hierarchies, while women are often relegated to lower statuses. Leadership roles have traditionally been associated with masculine traits. However, as technology advances and the workplace evolves, gender roles are constantly shifting as men and women engage with each other and adapt to changing cultural and social environments (Farrell, 2020). Despite significant progress toward achieving gender equality, complete gender equality in terms of wages or position levels in the workplace has not yet been realized. Most of the barriers to women’s progress stem from gender stereotypes. This situation can pose obstacles to women’s career advancement, encouraging gender bias in employment decisions and promoting self-limiting behaviors among women (Hentschel et al., 2019). Similarly, a conducted study substantiates the stereotypical belief that women and men have different characteristics, affirming the ingrained judgment that male traits are more closely associated with leadership requirements. The study findings indicate that men tend to believe they are better suited for positions of power in politics and as business executives (Kiser, 2015). Öngen and Aytaç (2013) investigated the attitudes and life values of university students toward gender roles. A study involving 324 university students revealed that there were distinct differences in the attitudes toward gender roles between male and female students. Women generally held more egalitarian views and were more open to non-traditional gender roles, while men tended to adhere to more conventional gender roles. The study also found a significant correlation between the attitudes of young individuals toward gender roles and their values, particularly in terms of their beliefs about gender roles within marriage and their support for egalitarian gender roles.

## 3 Gender inequality and women in the culinary profession

In the context of gender roles produced by traditional patriarchal order, the kitchen (along with the house in general) is associated with women (İnce, 2016). In traditional societies, men’s legitimate participation in cooking is linked to religion and rituals. Preparing the food to be sacrificed to the gods has always been the duty of men (Fürst, 1997). In this context, Szabo (2014) discusses the difference in roles between women and men in the culinary. When examining the gender-cooking relationship in line with social roles and responsibilities, it is observed that women cook with a great responsibility to please others, and to ensure the health and well-being of their loved ones. In addition, cooking for men can be seen as a leisure activity or a cooking show on special occasions. Cooking for men is a hobby, a culinary art performance, or a seduction strategy. These stereotypes that divide cooking into two for women and men are important in terms of understanding gender inequality and gender-based division of labor.

The number of women in the workforce started to increase after World War II. However, despite changes in women’s labor force participation, wages, and leadership positions continue to lag behind those of men (Kiser, 2015). Gender equality policies implemented in the European Union include the principles of equal pay and equal treatment. Comprehensive equality laws were developed in the 1970s to establish the rights of women as employees. As part of the reforms undertaken in the process of harmonization with the EU, Turkey has considered adding gender equality policies to its legal framework. In this context, discrimination based on gender was prohibited and the principle of equal treatment and “equal pay for the same or equivalent work” was adopted (Dedeoğlu, 2009). However, despite these efforts, gender discriminatory attitudes are still widespread in employment. Generally, women are subjected to various traditional gender roles that make them responsible for housework, childcare, elderly care, and family-related matters (Calvet et al., 2021). In the workplace, women are excluded due to the lack of organizational structures that are independent of gender. Additionally, women are excluded from organizational structures due to the belief that their bodily processes, such as pregnancy, breastfeeding, and menstruation, are not suitable (Acker, 1990). Unfortunately, this applies to the organizational structure of professional culinary that requires teamwork. In addition, since women take on different social roles simultaneously, they have to make more effort to develop themselves and receive promotions in the culinary (Kurnaz et al., 2018).

In numerous cultures, the overwhelming majority of women are responsible for cooking meals in their homes for their families. Meal organization has become one of the most important responsibilities of women (Reynolds, 2008). As the practice of cooking evolved from a necessity for survival to a highly specialized culinary art form catering to large groups, it was extremely uncommon to find female chefs until relatively recently (Bartholomew and Garey, 1996). Historically, professional culinaries have been dominated by men. In the fourteenth and fifteenth centuries, during times of war, men served as cooks in the army and developed a military-style hierarchy in the culinary (Platzer, 2011). In addition, it is known that in ancient Mesopotamia and Egypt, meals were served to nobles and dynasties in temples, and male cooks were responsible for preparing these meals (Swinbank, 2002). Male cooks can also be found in Mediterranean, European, and Ottoman palaces. The endurance and strength of men were likely reflected in military kitchens as well (Bozok and Korkmaz, 2019). Today, the military-based hierarchical organizational structure in professional culinary, which resembles the organization of a military unit, completes and confirms these historical facts and information about chefs and cooks.

Until the late 1900s, women were consistently barred from positions of authority and esteem in professional restaurant kitchens, particularly as head chefs (Druckman, 2010). In the 1970s and 1980s, women made up only about five to 10 % of those enrolled in culinary schools (Platzer, 2011). Although more women are now obtaining culinary degrees and some female chefs have gained recognition for their achievements, women still encounter significant challenges in attaining success in the culinary field (Harris and Giuffre, 2010). In professional culinary controlled by men, women are also subject to serious discrimination by being employed in low-status positions and facing obstacles to advancement. In addition, research has shown that female employees are particularly unwelcome in chef positions (İnce, 2016). Arnoldsson (2015) mentions a division in restaurant culinary where women are placed in the cold section and men in the hot section.

LaPointe (1992) states in her study that in hierarchical professional culinary, it is generally men who occupy the chef position. She attributes this to men’s control power over others and also to the perception of being more talented in physically demanding tasks. One could argue that men and women possess distinct traits, with qualities attributed to men being more closely associated with the demands of leadership roles. It can be argued that different perceptions of gender roles and stereotypes contribute to this situation (Kurnaz et al., 2018). An investigation into the issue of gender equality in the hospitality industry identified that employers’ entrenched attitudes and limited awareness about the subject were the primary obstacles to achieving equal opportunities for women (Burrell et al., 1997). Examining the challenges faced by women in the culinary industry is crucial for better understanding the operations and processes that perpetuate gender inequality in professions (Bozok and Korkmaz, 2019). There are studies in the literature investigating the challenges faced by women in professional culinary and the gendered attitudes they encounter (Harris and Giuffre, 2010; Haddaji et al., 2017; Çelik and Akar Şahi̇ngöz, 2018; Kurnaz et al., 2018; Tang and Baldwin, 2019; Taşpınar and Türkmen, 2020; Temizkan and Uslu, 2023).

Harris and Giuffre (2010) researched to examine how women who work as professional chefs perceive and react to stereotypes that suggest women are not capable of effectively leading in culinary fields, are overly emotional and are unsuitable for jobs dominated by men. The study found that, in the face of gender inequality in professional culinary where men are dominant, female chefs tend to emphasize their feminine traits rather than trying to act “like men” or hide their gender differences. Furthermore, the study discovered that female chefs often internalize various stereotypes associated with their gender in the culinary industry, such as the notion that they are not tough or commanding leaders. Instead, they prioritize being a skilled and proficient chef. Haddaji et al. (2017) conducted a study to identify the career challenges that women encounter in their pursuit of becoming head chefs. The findings revealed that female chefs encounter several barriers, including male dominance in the culinary industry, underestimation of their skills, and difficulties in achieving a work-life balance.

In a study conducted by Çelik and Akar Şahi̇ngöz (2018) on female chefs, most of the female chefs stated that discrimination occurred in hiring processes and they were employed in lower-level jobs than their skills warranted. A different study focusing on female chefs uncovered that possessing traits such as patience and adaptability toward teamwork can be advantageous for female chefs, while physical weakness is identified as their primary disadvantage. The most notable finding of this research was that female and male chefs are equivalent in terms of opportunities for promotion, pay, participation in management, and involvement in decision-making within the professional culinary field (Kurnaz et al., 2018).

In their research on female chefs, Tang and Baldwin (2019) discovered that female chefs encounter several obstacles, including physical capability, gender bias, limited chances for learning and advancement, and difficulties in balancing work and family responsibilities. Taşpınar and Türkmen (2020) conducted research to identify the obstacles that prevent female chefs from having long-term careers in the culinary industry. Their findings revealed that unfavorable working conditions within the culinary industry are the primary hindrance for women to advance their careers. The study also emphasized that the extended and flexible working hours prevalent in the culinary profession hurt the home life of women who have families.

Albors Garrigós et al. (2020) carried out a study that analyzed the success factors and barriers faced by chefs in their career paths, as well as the differences between genders, in Spain, France, and the United States. According to the study, the career of a chef demands a range of skills. Chefs noted that creating their brand and gaining public recognition is essential, and they emphasized that the working environment is challenging and competitive. It was found that female chefs obtain greater job satisfaction when they become self-employed. Temizkan and Uslu (2023) conducted research to identify the main reasons why women face barriers when working in the restaurant culinary industry. The study concluded that women encounter gender inequality during the hiring process, and pregnancy is the most common cause of discrimination toward women. Moreover, the profession’s physical and mental demands and its image are perceived as obstacles that deter women from entering the culinary profession. The study aimed to determine whether the primary obstacle for women in the culinary industry is gender inequality or reluctance due to the profession’s challenges.

## 4 Method

In this research, both quantitative and qualitative data collection methods were employed to determine the participants’ perceptions of gender inequality within the professional culinary in the tourism sector. In this context, in addition to closed-ended questions, open-ended questions were also posed to the participants to allow them to provide comments and express their thoughts in more detail.

The current study addresses gender inequality that emerges in the professional culinary within the tourism sector, which has significant universal implications in terms of social, cultural, and economic aspects. The study aims to shed light on the reasons behind gender inequality and, in particular, to identify the essential elements for female chefs to overcome gender bias and achieve a successful career while becoming effective leaders. In this context, the following hypotheses were formulated in the study:.

*H_1_*: The importance attributed situations causing gender inequality differ according to gender.

*H_2_*: The importance attributed situations causing gender inequality differ depending on previous experience working with a female chef.

*H_3_*: The importance attributed to the necessary qualities to be a good leader/head chef in the culinary differ according to gender.

*H_4_*: The importance attributed to the necessary qualities to be a good leader/ head chef in the culinary differ according to previous experience of working with a female chef.

*H_5_*: The importance attributed to factors influencing the support of female chefs’ careers differ according to gender.

*H_6_*: The importance attributed to factors influencing the support of female chefs’ careers differ according to previous experience of working with a female chef.

*H_7_*: The importance attributed situations causing gender inequality differ according to position.

*H_8_*: The importance attributed situations causing gender inequality differ according to the type of culinary one works in.

*H_9_*: The importance attributed to the necessary qualities to be a good leader/ head chef in the culinary differ according to position.

*H_10_*: The importance attributed to the necessary qualities to be a good leader/ head chef in the culinary differ according to the type of culinary one works in.

*H_11_*: The importance attributed to factors influencing the support of female chefs’ careers differ according to position.

*H_12_*: The importance attributed to factors influencing the support of female chefs’ careers differ according to the type of culinary one works in.

### 4.1 Population and sample of the research

The research population consists of students enrolled in culinary programs at the associate’s degree and gastronomy and culinary arts programs at the bachelor’s degree universities in Turkey. The research sample, on the other hand, comprises students who have previously worked in jobs related to the professional culinary of a tourism establishment and have agreed to participate in the study. The prior experience of participants in the culinary will enable them to provide answers and offer insights into questions related to gender inequality addressed within the scope of the research.

### 4.2 Data collection method and process

To obtain primary data on participants’ perceptions and thoughts regarding gender inequality in the culinary profession, a survey technique was used in the research. The survey used in the study was adapted from the survey used in (Farrell, 2020) study. The survey form consists of four parts. The first part includes statements to determine participants’ socio-demographic characteristics, characteristics related to working in the culinary/food and beverage department, and their thoughts on gender inequality. The second section of the survey form aims to determine the situations/topics that cause gender inequality in the culinary/food and beverage department. The third section includes statements regarding the qualities necessary to be a good leader/head chef in culinary, and the fourth section includes statements regarding the elements that are considered necessary to support the career development of female chefs. The scales used in the survey form were created according to a 5-point Likert scale ranging from “Not Important (Farrell, 2020)” to “Very Important (Ustabaş and Gülsoy, 2017).” In addition, open-ended questions were included to be able to address the opinions and thoughts of the participants in more detail regarding the issue.

The survey was conducted between January 3–27, 2023 to collect primary data on participants’ perceptions and thoughts regarding gender inequality in the culinary profession. While some data was collected face-to-face, the majority was collected through an online survey created using *Google Forms* to reach students in different cities more quickly. At the end of the data collection process, 396 surveys were completed. However, 24 surveys were excluded from the analysis due to missing data, especially because some participants indicated that they were not working in any job related to the research topic. As a result, the final number of surveys included in the analysis is 372.

### 4.3 Analysis of the data

SPSS statistical software was used to analyze the quantitative data obtained in the study. Frequency analysis was performed to evaluate participants’ socio-demographic characteristics, characteristics working in jobs related to the professional culinary/food and beverage department, and their thoughts on gender inequality. Validity and reliability analyses were conducted for the scales, and the arithmetic mean and standard deviation values of the statements were determined. In addition, independent samples t-test and one-way analysis of variance (ANOVA) were performed to identify group differences related to the scales. The significance level used in the analyses was set at 0.05.

The data obtained from open-ended questions obtained in the study were analyzed using the descriptive analysis method. According to the descriptive analysis approach, the obtained data is summarized and interpreted according to previously determined themes. It is possible to organize the data according to the themes revealed by the research questions or the questions/dimensions used in the data collection process (Yıldırım and Şimşek, 2011). In this study, the themes were generated from the research questions for the analysis of qualitative data. The analysis of comments from participants, which included their thoughts and perceptions on the issue, was conducted without the use of any analysis program because the data was not large enough to require the use of an analysis program. To prepare the qualitative data for analysis, the expressions related to their opinions were collected in a separate Word document by examining the survey forms in which the participants expressed their views. To ensure *transferability (external validity)* (Pandey and Patnaik, 2014), *a detailed description* of the “data collection process, instrument, and study group” related to the obtained data was provided in the study. The themes, categories, and codes obtained from the analysis of data were created. To demonstrate the *consistency (reliability)* of the data, the themes, categories, and codes were subjected to *peer review* by sharing the raw data (Pandey and Patnaik, 2014). In addition, quotations from the participants’ statements were used to reflect their detailed opinions on the issue.

## 5 Findings

### 5.1 Quantitative findings

Validity and reliability values for the scales used in the study were determined using the Kaiser-Meyer-Olkin Measure of Sampling Adequacy (KMO) and Cronbach’s Alpha coefficient (Table 1). According to the results, the KMO value of the scale used to identify situations/topics causing gender inequality in the culinary/food and beverage department was 0.834 and the Cronbach’s Alpha coefficient was 0.801. The KMO value of the scale containing statements about the necessary qualities to be a good leader/head chef in a culinary was 0.924 and the Cronbach’s Alpha coefficient was 0.895. The KMO value of the scale containing statements about the elements needed to support the career development of female chefs was 0.891 and the Cronbach’s Alpha coefficient was 0.817. The obtained results indicate that the validity and reliability of the scales used in the study were ensured.

**Table 1:** The validity, reliability, mean, and standard deviation values of the scales.

Statements	KMO Measure of Sampling Adequacy	Cronbachs Alpha	Mean	Standard Deviation
*The degree of importance of situations that cause gender inequality in a professional culinary work environment (GI)*	0,834	0,801		
The general cultural level of employees			4,24	0,98
The dominant gender in the work environment.			3,16	1,42
The approach of culinary managers.			**4,59**	0,73
The approach of managers			4,54	0,79
Long working hours			4,0	1,20
Supportive attitude toward maternity leave			4,33	1,04
Non-transparent promotion channels			4,21	1,17
Elimination of male-dominated structure			4,08	1,25
Equal pay			**4,58**	0,88
Shift arrangements			4,49	0,84
	Overall mean		4,23	
*Importance level of the required qualities to be a good leader/head chef in a culinary (GL)*	0,924	0,895		
Having an inspiring personality as a leader/chef			4,59	0,73
The ability to represent the team fairly.			**4,68**	0,61
Professional approach to team members.			4,63	0,68
Being decisive (in tasks, task distribution, etc.)			4,64	0,68
Ensuring unity among team members			4,65	0,65
Treating employees thoughtfully			**4,69**	0,61
Being passionate			4,57	0,73
Multitasking			3,98	1,07
Stamina			4,47	0,73
Flexibility			4,24	0,85
	Overall mean		**4,51**	
*The importance ranking of factors to support/advance the careers of female chefs in the industry (CFC)*	0,817	0,891		
Facilitating attitude toward paternity leave			4,20	0,95
Ensuring job safety			**4,62**	0,74
Gender balance in jury panels for chef and culinary competitions			4,26	1,08
Supportive life partner			4,51	0,85
Equal pay for equal work between women and men			**4,71**	0,69
Presence of female role models in the industry			4,48	0,86
The gender of the head chef in a workplace			**2,84**	1,56
Flexible working conditions			4,17	0,9
Easy access to information on maternity leave/rights			4,45	0,84
	Overall mean		**4,25**	

Bold values in the “Mean” column are the values with the highest and lowest mean.

The findings related to the socio-demographic characteristics of the participants are presented in Table 2. Based on the results of the frequency analysis of the socio-demographic characteristics of the participants, it can be seen that the majority of the participants are women (60.5%) and in the age range of 18–23 years old (92.7%). When the distribution of the participants according to their class is examined, it is seen that 31.7% are in the 3^rd^ class and 29.8% are in the 2^nd^ class. Furthermore, the vast majority of the participants (89.8%) are enrolled in the first education type.

Table 3 presents the frequency analysis results regarding the participants’ working status in a culinary-related job. According to Table 3, it has been determined that the majority of the participants work in a full-time job (73.1%) and in a hotel culinary (54.6%). The majority of the participants worked as sous chefs, busboys, and waiters/waitresses (52.2%) and had worked for 90 days or less (40.6%). The expected internship duration for students in culinary/gastronomy and culinary arts programs in Turkey is 90 days. Therefore, it can be inferred that the majority of the participants are likely to be internship students. When asked if they had worked with a female chef before, 52.7% of the participants answered no, while 47.3% answered yes.

**Table 2:** Frequency analysis for socio-demographic characteristics.

	*N*	%		*N*	%
*Gender*			*Age*		
Female	225	60,5	18–23 years	345	92,7
Male	147	39,5	24 years and above	27	7,3
*Class*			*Education type*		
1. Class	51	13,7	1st education	334	89,8
2. Class	111	29,8	2nd education	38	
3. Class	118	31,7	Total	*N*	%
4. Class	92	24,7		372	100

**Table 3:** Frequency analysis of working in a job related to the culinary.

	*N*	%		*N*	%
*Working status in the culinary*			*The period of working in the culinary*		
Full time	272	73,1	90 Days or Less	151	40,6
Part-time	100	26,9	3–6 Months	122	32,8
*Last worked culinary type*			6–12 Months	55	14,8
Fine dining restaurant	35	9,4	1 Year and above	44	11,8
Ethnic restaurant	11	3	*Job description in the last worked culinary*		
Hotel culinary	203	54,6	Head Chef	11	3
Catering	12	3,2	Sous Chef	16	4,3
Cafe	42	11,3	Chef de Partie	38	10,2
Casual restaurant	35	9,4	Chef apprentice/busboys/waiter/waitress	194	52,2
Institutional culinary	15	4	Trainee	113	30,4
Pastry/bakery	19	5,1	*Previous work experience with a female chef*		
*Total*	372	100	Yes	176	47,3
			No	196	52,7

Table 4 presents the results of the frequency analysis on the participants’ thoughts on gender inequality. 59.7% of the participants answered “yes” to the question “Do you think there is gender inequality in the culinary profession in Turkey?” when asked if they experienced gender inequality issues in the last worked culinary, 47.8% answered no, and 37.4% answered yes. While 86.6% of the participants stated that there is no course or topic related to gender inequality in their educational program, 67.2% expressed the necessity of having a course on gender inequality in the program curriculum.

**Table 4:** Frequency analysis on gender inequality.

	*N*	%		*N*	%
*The perception of gender inequality in the culinary profession in Turkey*			*The existence of a course on gender inequality in the program being studied*		
Yes	222	59,7	Yes	22	5,9
No	83	22,3	No	322	86,6
I am undecided/I have no opinion	67	18	I am undecided/I have no opinion	28	7,5
*Perception of experiencing gender inequality issues in the last worked culinary*			*Thoughts on the necessity of having a course on gender inequality in the program curriculum*		
Yes	139	37,4	Yes	250	67,2
No	178	47,8	No	75	20,2
I am undecided/I have no opinion	55	14,8	I am undecided/I have no opinion	47	12,6
*Total*	372	100	*Total*	372	100

Table 1 provides the results of the validity and reliability analyses of the scales used in the study, the mean and standard deviations of the scale items, and the overall mean of the scales. The overall average of the scale related to “Situation That Causes Gender Inequality in a Professional Culinary Environment” is 4.23. The statements with the highest averages on this scale are “The approach of culinary managers (4.59)” and “Equal pay (4.58),” indicating that the participants consider the approach of managers toward female and male employees and the necessity of equal pay for male and female employees as important/very important. The overall average of the scale related to “Necessary Qualities to Be a Good Leader/Chef in a Culinary” is 4.51. The statements “Treating employees thoughtfully (4.69)” and “The ability to represent the team fairly (4.68)” are the statements with the highest level of importance. The overall average for the “Important Factors for Supporting/Advancing the Careers of Female Chefs in the Industry” scale is 4.25. The statements with the highest importance ratings on this scale are “Equal pay for equal work for women and men (4.71)” and “Ensuring job safety (4.62).” It is also possible to say that the gender profile of the head chef in a workplace is seen as of little important or moderately important in supporting the career of female chefs (2.84). This indicates that the participants expect equal treatment of employees regardless of gender in the industry. According to the statement, the gender that dominates the work environment is relatively less important than other factors that contribute to gender inequality in professional culinary (3.16). This can be interpreted as the fact that the person who causes gender inequality may not always be of the opposite gender.

Table 5 presents the results of independent samples t-tests conducted to test the research hypotheses. The aim was to determine whether there was a significant difference between participants’ gender, and previous experience working with a female chef, and situations causing gender inequality in the culinary/food and beverage department, the essential qualities required to be a good leader/head chef in a culinary, and the factors required to support female chefs’ career development. According to Levene’s test for equality of variances, results based on the assumption of unequal variances were considered for variables with significance values below 0.05. For variables with significance values above 0.05, results based on the assumption of equal variances were considered.

**Table 5:** Independent samples *t*-test results.

		*n*	Mean	Levene’s test for equality of variances		P (2-tailed)	Accepted/ Rejected status
				*F*	*p*		
*Gender*							
GI	Female	225	4,40	13,556	,000	,000	H_1_ Accepted
	Male	147	3,96				
GL	Female	225	4,57	7,870	,005	,006	H_3_ Accepted
	Male	147	4,42				
CFC	Female	225	4,40	19,002	,000	,000	H_5_ Accepted
	Male	147	4,01				
*Previous work experience with a female chef*							
GI	Yes	176	4,17	2,185	,140	,113	H_2_ Rejected
	No	196	4,27				
GL	Yes	176	4,49	,440	,508	,460	H_4_ Rejected
	No	196	4,53				
CFC	Yes	176	4,18	5,324	,022	,042	H_6_ Accepted
	No	196	4,31				

Upon examining the independent samples t-test results, a significant difference has been found (*p*-2 tailed <0.05) between gender and situations causing gender inequality in the culinary/food and beverage department (H_1_), the necessary qualities to be a good leader/head chef in a culinary (H_3_), and the necessary elements to support the career development of female chefs (H_5_). Accordingly, female participants attributed higher importance to situations causing gender inequality in the culinary/food and beverage department, the necessary qualities to be a good leader/head chef in a culinary, and the necessary elements to support the career development of female chefs compared to male participants.

No significant difference has been found between the previous work experience with a female chef and situations causing gender inequality in the culinary/food and beverage department (H_2_) and the necessary qualities to be a good leader/head chef in a culinary (H_4_) (*p*-2 tailed>0.05), but a significant difference has been found between the necessary elements to support the career development of female chefs (H_6_) (*p*-2 tailed<0.05). Participants who have not previously worked with a female chef attributed higher importance to the necessary elements to support the career development of female chefs compared to those who have worked with a female chef before.

Table 6 presents the results of one-way analysis of variance conducted to test the research hypotheses. For variables with significance values below 0.05 in the homogeneity test of variances under ANOVA, the results were evaluated based on the assumption of unequal variances. Therefore, Brown-Forsythe or Welch test values were examined for significance values (Antalyalı, 2010). For variables with significance values above 0.05, the results were considered based on the assumption of equal variances.

**Table 6:** ANOVA results.

		n	Mean	Test of homogeneity of variances	F	p	Difference	Accepted/ Rejected status
*Job title (Position)*								
GI	(1) Head Chef	11	4,22	,354	4,146	,003	3–5	H_7_ Accepted
	(2) Sous Chef	16	4,26					
	(3) Chef de Partie	38	3,92					
	(4) Ch.App./Busboy/Waiter	194	4,19					
	(5) Trainee	113	4,38					
GL	(1) Head Chef	11	4,49	,007	2,917	,029	3–4 4–5	H_9_ Accepted
	(2) Sous Chef	16	4,66					
	(3) Chef de Partie	38	4,25					
	(4) Ch.App./Busboy/Waiter	194	4,52					
	(5) Trainee	113	4,56					
CFC	(1) Head Chef	11	4,32	,142	2,120	,078		H_11_ Rejected
	(2) Sous Chef	16	4,22					
	(3) Chef de Partie	38	4,00					
	(4) Ch.App./Busboy/Waiter	194	4,25					
	(5)Trainee	113	4,34					
*Culinary type*								
GI	(1) Fine Dining Restaurant	35	4,19	,188	2,105	,042	3–6	H_8_ Accepted
	(2) Ethnic Restaurant	11	4,00					
	(3) Hotel Culinary	203	4,32					
	(4) Catering	12	4,30					
	(5) Cafe	42	4,1					
	(6) Casual Restaurant	35	3,9					
	(7) Institutional Culinary	15	4,19					
	(8) Pastry/Bakery	19	4,05					
GL	(1) Fine Dining Restaurant	35	4,43	,534	1,142	,336		H_10_ Rejected
	(2) Ethnic Restaurant	11	4,56					
	(3) Hotel Culinary	203	4,56					
	(4) Catering	12	4,55					
	(5) Cafe	42	4,49					
	(6) Casual Restaurant	35	4,42					
	(7) Institutional Culinary	15	4,58					
	(8) Pastry/Bakery	19	4,27					
CFC	(1) Fine Dining Restaurant	35	4,18	,550	1,554	,148		H_12_ Rejected
	(2) Ethnic Restaurant	11	4,37					
	(3) Hotel Culinary	203	4,33					
	(4) Catering	12	4,21					
	(5) Cafe	42	4,18					
	(6) Casual Restaurant	35	4,01					
	(7) Institutional Culinary	15	4,22					
	(8) Pastry/Bakery	19	4,11					

The study found a significant difference between the job title (position) of the participants in their last workplace and the situations causing gender inequality in the culinary/food and beverage department (H_7_) and the necessary qualities to be a good leader/head chef in the culinary (H_9_) (*p* < 0.05). However, no significant difference was found between the job title and the factors necessary to support the career development of female chefs (H_11_) (*p* > 0.05). To determine which groups the significant differences between the groups came from, the Gabriel test was conducted. According to the Gabriel test results, the participants who worked as interns attributed higher importance to the situations causing gender inequality in the culinary/food and beverage department than those who worked as chef de partie. Those who worked as assistant chef/server/trainee attributed greater importance to the necessary skills to be a good leader in the culinary than those who worked as chef de partie.

Significant differences were found only between the participants who last worked in culinary type and the situations causing gender inequality in the culinary/food and beverage department (H_8_) (*p* < 0.05). No significant differences were found between the necessary qualities to be a good leader/head chef in a culinary (H_10_) and the elements required to support the career development of female chefs (H_12_) with the culinary type (*p* > 0.05). The Gabriel test was conducted to determine which groups were responsible for the significant differences between the groups. According to the results of the Gabriel test, participants working in hotel culinary contributed more importance to situations causing gender inequality in the culinary/food and beverage department than those working in restaurants.

### 5.2 Qualitative findings

In the study, open-ended questions were included to obtain detailed information on qualitative findings and allow participants to express their views, thoughts, and experiences on the issue. The following research questions were created to provide a context for participants’ thoughts and experiences.

*RQ_1_*: What are the perceived problems related to gender inequality in the culinary profession?

*RQ_2_*: What are the thoughts and experiences regarding working with a female chef?

*RQ_3_*: What are the perceived qualities necessary to be a good leader/head chef in a culinary?

*RQ_4_*: What are the important factors believed to support the career development of female chefs?

The number of participants who shared their views and experiences regarding gender inequality in the professional culinary is 200. Table 7 includes the theme “Issues related to gender inequality in the professional culinary” and its categories and codes. The theme of gender inequality in the culinary profession has been categorized into four categories: “*Negative Attitudes Toward Female Employees*,” “*Negative Attitudes Toward Male Employees*,” “*Factors Leading to Gender Inequality Against Women*,” and “*Other Issues*.”

**Table 7:** Gender inequality issues in culinary profession.

Codes	*N*	%
*Category 1. Negative attitudes toward female employees*		
Assigning women with simpler tasks and considering them as inadequate	66	33
Prejudices that women will fail	21	10,5
Belittling, demeaning, and oppression of women	21	10,5
Sexual expectations/abuse, harassment, and rape toward female employees	18	9
Resistance to hiring women/having female staff in the culinary	17	8,5
Perception of women as weak	14	7
Women not being given leadership positions/advancement	13	6,5
Psychological pressure-mobbing	11	5,5
Women being kept in the background	8	4
Not giving women the right to speak up and not respecting their ideas	6	3
Failure to transfer professional knowledge to female employees	1	0,5
Comparing female employees with male employees.	1	0,5
*Category 2. Negative attitudes toward male employees*		
Employing male employees under more strenuous working conditions than female employees	8	4
More privileged treatment of female employees	8	4
Male employees being oppressed or perceived as insignificant	5	2,5
Female employees being treated more delicately	4	2
*Category 3. Situations that lead to gender inequality against women*		
The low number of female employees in the culinary	1	0,5
Female employees being mothers	1	0,5
Women not being able to develop themselves	1	0,5
The general cultural level of the employees	1	0,5
Family attitudes	1	0,5
*Category 4. Other issues*		
Unfair distribution of work/tasks	27	13,5
Men are considered superior in terms of physical strength	27	13,5
Male dominance due to patriarchal social structure	22	11
Inequality in salaries	7	3,5
Poor management and abusive behavior by managers	5	2,5
Male employees do not want female employees in the culinary because they cannot speak freely	5	2,5
Challenging working conditions	5	2,5
The perception that a job/profession is not suitable for women	5	2,5
Long working hours	3	1,5
The culinary is an environment that requires patience	2	1
A stressful job	2	1
Discrimination/Inequality	2	1

When Table 7 is examined, it is concluded that the participants do not perceive gender inequality only toward women. In some cases, participants expressed the view that the tolerance shown to female employees is not shown to male employees. It was determined that the most disturbing situation for the participants in the category of negative attitudes toward female employees was giving simple tasks to female employees. The participants also expressed discomfort with the presence of female employees in the culinary department, especially in the main section, where physically demanding tasks are required. They mentioned that *female employees are not able to perform such tasks* and that *their presence hinders the use of vulgar language*. They also mentioned that female employees are subjected to *verbal and physical harassment in the workplace*, and are *given relatively unimportant* and *simple tasks such as cleaning or preparation, which is seen as a sign of not wanting to hire them in the culinary department. Unequal job and income distribution, wage inequality, difficult working conditions, and discrimination* were also mentioned by participants as negative situations for both female and male employees. Below are some quotes from participants (***P***) regarding the issue.

*P14. If you are a woman in the culinary, instead of teaching you something they think you cannot do, they ignore you and give you simple tasks.*

*P16. At my last workplace, only one female employee was able to start working, but there was no long-term and efficient working system. Even when we suggested the idea of hiring a female staff member, we faced reactions from other employees.*

*P74. Some people belittle women by saying that they cannot do certain jobs or tasks and ask why female employees are being hired.*

*P77. Because in Turkey, it is generally looked down upon for a woman to want to work in big culinaries. The reason is the supposed weakness of women. In most of the places where I worked, despite being more knowledgeable and skillful than my male colleagues, this was generally overlooked.*

*P101. In the main section, which is the most active and where the employees constantly move, female employees can be subjected to harassment under the guise of “work.”*

*P106. Violating boundaries, not respecting opinions, assigning menial tasks, and using vulgar language.*

*P165. In hotel culinary and the industry in general, men are often burdened with more work, while women work in more positive conditions. There is unequal treatment in terms of workload. Generally, men have a heavier workload, while women work in easier conditions, such as in the patisserie.*

*P186. When forming culinary teams, women are often not wanted in the team and men are assigned the more difficult tasks in the culinary.*

*P199. Men are assigned heavier tasks while women are given cleaning-oriented tasks. Women are treated more optimistically during entry and exit times.*

*P240. Women are kept in the background in recruitment and men are given priority when it comes to advancement, which affects salaries as well.*

*P269. There are difficulties in every area and chefs do not want women to learn anything. I have worked in many places and have heard things like “You are a woman, you cannot handle heavy work, the culinary is tough, you are delicate,” and chefs do not want to pass on their knowledge to women.*

Table 8 includes the theme “Thoughts and experiences about working with a female chef” and the categories and codes related to this theme. The number of participants who shared their thoughts and experiences about working with a female chef in the culinary department is 88.

**Table 8:** Thoughts and experiences regarding working with a female chef.

Codes	*N*	%
*Category 1. Positive judgments*		
Being more understanding	19	21,59
Meticulous, careful, detail-oriented	6	6,81
Happy and respectful work environment	6	6,81
To be clean and tidy	4	4,54
To be protective, nurturing	4	4,54
Emergence of beautiful works	3	3,40
Serious work environment	3	3,40
Being able to pull things together	2	2,27
Female solidarity	2	2,27
Having good and instructive professional knowledge	2	2,27
Calm and safe environment	2	2,27
Diligence	2	2,27
Being kind	2	2,27
Skillful	2	2,27
Enjoyable	2	2,27
Working with a male chef and a female chef is the same	2	2,27
Being more interested	1	1,13
Having an esthetic perspective	1	1,13
Being able to be a role model	1	1,13
Being more conscious and responsible	1	1,13
Maternal instinct	1	1,13
Being authoritarian	1	1,13
*Category 2. Negative judgments*		
Negative behaviors toward peers of the same gender	5	5,68
Competition and jealousy	3	3,40
Making emotional decisions	2	2,27
Negative behaviors due to seniority	1	1,13
Weakness in self-control	1	1,13
Being too selfish	1	1,13
Working until late is a problem	1	1,13
Not being as advantaged as men in terms of advancement	1	1,13
Negative attitudes toward female chefs	1	1,13
Talking too much	1	1,13
Interfering in everything	1	1,13
Being tense because of being criticized despite doing more work than male counterparts	1	1,13
Dominant	1	1,13
Lack of respect toward female chefs	1	1,13
Physically inadequate	1	1,13

The codes obtained from the responses of the participants regarding their thoughts and experiences about “Working with a female chef in the culinary department” were categorized as “*Positive Judgments*” and “*Negative Judgments*.” Among the positive judgments, the most emphasized topic is that female chefs are more understanding. In addition, positive qualities such as *being organized, maternal, skillful, and responsible for cleanliness and order* have been highlighted for female chefs. It is noteworthy that these values attributed to female chefs are often associated with the roles socially assigned to women. These findings also correspond with the participants’ views that the characteristics of patriarchal societal structure are reflected in the work environment. Among the negative aspects of working with a female chef, participants mentioned personal traits such as *negative behavior toward female colleagues, emotionalism, and selfishness, as well as work-related issues such as the inability to keep up with long working hours, not being as advantaged as male employees/chefs in terms of advancement, lack of sufficient respect, and the creation of a stressful work environment due to the tensions* caused by these situations. Quotations from the participants’ thoughts and experiences about working with a female chef are provided below.

*P1. I would like to work and get experience with a female chef because I believe that women have a more aesthetic perspective.*

*P14. My female chef was teaching me something with interest and taking me under her wing because the male chefs were ignoring me.*

*P34. I had a very nice, enjoyable, and serious experience, and the approach of the other chefs was very good.*

*P78. She had good a understanding as a woman, but the pecking order could disappear due to familiarity and lack of self-control in communication, of course, this may be due to personal characteristics.*

*P99. Thanks to our chef’s maternal attitude, I was able to work in a less stressful work environment.*

*P123. I do not think that female chefs can manage crises better because they make more emotional decisions.*

*P148. There was no problem in terms of the work we did, but leaving late could be a problem for her family.*

*P246. I think that women are more careful, more instructive, and more hardworking compared to men.*

*P260. Female chefs do not want to give experience to female colleagues. There are also quite a few who apply mobbing.*

*P265. She was doing more work than the male chef, but she was working in a tense atmosphere because she was criticized more, and it was causing tension for everyone. While the male chef was respected, the same respect was not shown to the female chef.*

Table 9 includes the theme of “Qualities Required to be a Good Leader/Head Chef in the Culinary” and the corresponding codes related to this theme. Twenty-six participants expressed their views on the qualities of a good leader/head chef in the culinary.

**Table 9:** Necessary qualities for being a good leader/head chef in the culinary.

Codes	*N*	%
Should be equal and fair	10	38,46
Should be understanding	7	26,92
Should be directive	4	15,38
Should be equipped	3	11,53
Should be experienced and knowledgeable	3	11,53
Should be solution-oriented	3	11,53
Should be authoritative	2	7,69
Should be calm and kind-hearted	2	7,69
Should protect the rights of employees	2	7,69
Should love their job	1	3,84
Should be innovative	1	3,84
Should not be solely focused on financial gain	1	3,84
Should be fun	1	3,84
Should adhere to ethical and moral principles	1	3,84
Should be professional	1	3,84
Should be responsible	1	3,84
Should set an example	1	3,84

When asked about the characteristics of a good leader/ head chef, the participants generally referred to job-related qualities. Therefore, they emphasized the necessity for a leader/head chef to show equal and fair treatment to all employees without any discrimination. In this context, they also highlighted the importance of a good leader/head chef to protect the rights of employees and be professional. *Solution-oriented approach, experience, equipment, knowledge, authority,* and *innovation* are also the qualities that are sought after in a good leader/head chef related to their knowledge and skills about the job. Additionally, it can be stated that personality traits such as *calmness, kindness, and being fun* are also among the personality traits that a good leader/ head chef should possess. Quotations from participants’ statements on the subject are included below.

*P6. They should be directive and prioritize the rights of their employees,* etc.

*P86. In the culinary, the head chef should be very understanding. They should be fair to everyone and have developed themselves in almost every aspect.*

*P143. Chefs should be innovative.*

*P162. They should be authoritative and objective. They should find quick solutions to problems.*

*P204. I think the most important thing is to be considerate toward their team members, stand behind them, and explain their mistakes correctly and properly, not in a bad way.*

*P273. Being able to set an example with their behavior and actions in addition to their knowledge.*

*P334. Having sufficient knowledge and experience, being supportive and positive in their attitude and behavior toward their employees.*

*P336. A chef or senior employee should take a close interest in their employees and try to solve any problems. They should be impartial and separate their emotions from work.*

Table 10 includes the theme “Important factors in supporting the career of female chefs” and corresponding codes. Twenty participants expressed their opinions about the important factors in supporting the careers of female chefs.

**Table 10:** Important factors in supporting the careers of female chefs.

Codes	*N*	%
*Category 1. Internal factors*		
Women should have more self-confidence	2	10
Women should be strong/persistent	2	10
Women should be courageous enough to take risks	1	5
*Category 2. External factors*		
Removing barriers to women’s advancements	3	15
More job opportunities should be given to female chefs	2	10
Providing a peaceful work environment	2	10
Changing the perspective toward female chefs	1	5
Flexibility should be provided during leave and working hours	1	5
Appreciating the work done by women	1	5
Male dominance should not be given priority in intern hiring	1	5
Supportive awards should be given	1	5

The codes obtained from the responses of the participants regarding their thoughts and experiences about “Important factors in supporting the careers of female chefs” were categorized as “*Internal Factors*” and “*External Factors*.” *External factors* refer to the expectations from managers/employers in supporting the careers of female chefs. *Internal factors,* on the other hand, emerged as a result of criticism/self-criticism regarding the aspects that female chefs need to develop/strengthen in supporting their careers. According to Table 10, the factors that are attributed important in supporting the careers of *female chefs include removing the obstacles to advancement for female chefs, boosting the self-confidence of women, providing more job opportunities for female chefs, empowering women to be strong, and creating a peaceful working environment*. The findings suggest that the lack of self-confidence among female employees plays a significant role in hindering the career advancement of female chefs, which could be due to both personality traits and oppressive attitudes and behaviors in the workplace. The findings related to promotion issues and a peaceful working environment are consistent with the problems of gender inequality. Therefore, it is possible to say that gender inequality in the culinary department will hurt the careers of female chefs. The following are some quotes related to the factors that female chefs consider important in supporting their careers.

*P87. Changing the perceptions of female employees by society and the industry.*

*P163. I think women need to be stronger and more decisive to have more of a voice in the culinary.*

*P165. If they want to see themselves as better and more successful in this industry, they should make themselves mentally and physically strong enough to be equal to men.*

*P184. Above all, they need to believe in themselves.*

*P218. The elimination of disruptive behaviors, providing a peaceful work environment, removing barriers to advancement, and having a supportive attitude.*

## 6 Results and discussions

The culinary profession has been dominated by men worldwide for a long time, but it is a rapidly developing field that is increasingly attracting the interest of women. However, gender inequality in the culinary profession is a significant problem that can hinder the potential and talents of many female chef candidates, reflecting gender inequality in society and leading to a male-dominated work environment. The quantitative findings of the research revealed differences in the importance attributed to gender inequality among participants based on demographic characteristics (gender) and work-related situations (position and type of culinary worked in). The findings obtained from the open-ended questions of the research provided insights into the reasons or circumstances behind the differences in the attributed importance. In this context, it can be stated that female participants perceive more gender inequality and attribute more importance to gender inequality compared to male participants (H_1_), and they suffer more from gender inequality. However, it can be interpreted that participants working as interns attribute more importance to/experience/witness gender inequality (H_7_), despite these discriminatory elements being a significant issue at every level. It is suggested that interns being perceived temporarily in the work environment and being perceived as the lowest-level position lead them to experience more gender inequality. Regarding the type of culinary worked in, it was found that the importance attributed to gender inequality is higher among hotel employees (H_8_). The higher perception of these inequality elements in hotel culinary is considered noteworthy due to the higher number of participants working in this type of culinary compared to others. Among the situations related to gender inequality emphasized by the participants in their responses to open-ended questions, it is highlighted that men have more job opportunities and are in a superior position to their female colleagues in terms of promotions. Additionally, prejudices against the employment of women in the kitchen and men receiving higher salaries than their female colleagues are important indicators of gender inequality in the sector. The gender roles and gender inequality attributed to women and men by society can cause gender inequality in the professional culinary. Based on the research findings, it has been determined that in the culinary profession, gender inequality affects women more significantly, although a small number of male employees have also complained about gender inequality. This allows for the generalization that gender inequality predominantly impacts female employees in the culinary profession. On the other hand, it is possible to express that women’s exposure to physical or psychological harassment at work is the most disturbing aspect of gender inequality.

The existence of gender inequality in the work environment causes problems for female employees in leadership positions in the culinary as well. In this context, the quantitative findings of the research indicate that female participants attribute more importance to the necessary qualities to be a good leader/chef in the culinary compared to male participants (H_3_). According to this result, it can be inferred that female chefs may need to exert more effort to establish themselves as leaders. The responses to the open-ended questions in the research also support this interpretation. A significant number of participants not only expressed support for female chefs taking on leadership roles but also mentioned some challenges they might face. Issues such as the superiority of male employees in promotions, the advantages bestowed upon men by patriarchal societal features, the difficulty for women in balancing work and family life, the lack of serious consideration for female leaders, and conflicts with female colleagues are among the important problems raised by participants that contribute to gender inequality. The research identified differences between the necessary qualities to be a good leader/chef and positions (H_9_). Accordingly, employees at every level attributed high importance to the qualities required to be a good leader/chef, with those in the sous chef position assigning the highest level of importance. When evaluating differences between positions, it is noteworthy that differences emerge among lower-level employees. This finding suggests that lower-level employees may need more support from their superiors. Moreover, the responses to the open-ended questions in the research support this conclusion, as female participants mentioned being directed toward more burdensome or simple tasks during their internships, not being taught the intricacies of the profession, and expressing that their ideas were not valued.

In the professional culinary, despite gender inequality, the increasing interest of women in the profession and the growing number of successful female chefs are paving the way for women to take on leadership roles in the field. Female chefs can take on leadership roles by opening their restaurants and serving as head chefs in large restaurants. Female chefs also demonstrate their strong presence in the industry by performing successfully in cooking competitions. Some famous female chefs who have successfully taken on leadership roles in the industry include *Clare Smyth* with her restaurant *Core* in London (The World’s 50 Best, 2021), *Dominique Crenn* with her restaurant *Atelier Crenn* in San Francisco (The World’s 50 Best, 2021), and *Ana Ros* with her restaurant *Hiša Franko* in Slovenia (The World’s 50 Best, 2021), all of which are included on The World’s 50 Best Restaurants list. The leadership roles of female chefs also present an opportunity for solving the problem of gender inequality in the industry. Female chefs’ leadership roles can increase workforce variety in the industry and serve as a source of motivation for other women to pursue this profession. In addition, the successful performance of female chefs in leadership roles can contribute to raising awareness of gender inequality in the work environment. However, while most participants support female chefs taking on leadership roles, they also express some of the challenges that female chefs may face, such as the promotion advantage of male employees, advantages that patriarchal society gives to men, difficulty balancing work and family life for women, lack of respect for female leaders, and conflicts with their female peers.

Another topic investigated within the scope of the current study is the important factors for supporting the careers of female chefs. It is believed that supporting the careers of female chefs in the culinary is crucial in creating a fairer and more equal work environment. In this context, female participants have attributed higher importance to the important factors for supporting the careers of female chefs (H_5_). A difference has been identified between the situation previous experience of working with a female chef and solely the importance attributed to the important factors for supporting the careers of female chefs (H_6_). In light of these results, it is believed that female employees view their chefs as role models, which could enhance their motivation for achievement. Additionally, it can be inferred that female chefs are willing to support their female peers. No significant difference has been found between previous experience of working with a female chef and the importance attributed to gender inequality (H_2_) and the importance attributed to the qualities needed to be a good leader/chef (H_4_). This suggests that female chefs are perceived by participants as not engaging in behaviors that would lead to the perception of gender inequality and that they exhibit a supportive attitude in being a good leader/chef. However, the findings to the open-ended questions reveal mixed opinions on this matter. While some participants express concerns about potential workplace jealousy among women, others emphasize that their feelings, expectations, and desires are better understood by their female peers. Nevertheless, the findings to the open-ended questions in the research highlight that empathy prevails over jealousy. Participants have emphasized the need to remove the barriers that hinder female employees from getting promoted and increase job opportunities for women, as well as the importance of building up the self-confidence of women in the industry, to support the careers of female chefs.

## 7 Implications

It is considered that evaluating gender inequality in the culinary profession only as an issue affecting employees provides an incomplete perspective on the subject. Problems stemming from gender inequality can also have negative effects on workforce variety and workplace culture in the culinary, which can ultimately harm businesses. Experiencing gender inequality can negatively impact female chefs’ job satisfaction and lead to them leaving the profession, which can result in the loss of talented female chefs and negatively affect workforce variety. To overcome these issues, employers can adopt fairer and more equal hiring, promotion, and salary policies and avoid tolerating gender inequality. In addition, encouraging women to take on more leadership roles in the industry can promote greater female participation in the workforce and strengthen their presence in the industry. Thus, it may be possible for women to have a greater presence in the profession and for different perspectives and talents to be brought into the industry. This situation can help encourage innovations in the sector and give businesses a competitive advantage. Employers can also take specific measures to support and advance the careers of female chefs. They can invest more in training and development opportunities for female employees to advance their careers in the culinary profession. To support and advance the careers of female chefs in the culinary profession, the following suggestions can be made:

- Businesses can create awareness to emphasize and support the leadership potential of female chefs. This can enable women to showcase their talents and take on more leadership positions, and can also increase awareness of gender inequality throughout society.
- Businesses can create mentorship programs for female employees to teach the necessary skills for progressing in leadership positions. This could be an important solution, especially for female employees to have leadership and management skills in a male-dominated work environment.
- Equal conditions and opportunities should be provided to both female and male employees. Equal pay, working hours, workload, and promotion opportunities can be evaluated within this scope.
- Businesses can offer flexible working hours as a solution to allow talented female employees to balance their careers and family lives. This could increase the commitment of female employees to the business and enable them to progress in their careers.
- An open communication culture that encourages respect for each other’s ideas and rights without gender inequality can be established.
- Prejudices and discrimination that female chefs may encounter in the workplace should be prevented. Employers can support female chefs by not tolerating gender inequality in the workplace.
- Employers can provide networking opportunities such as meeting other chefs and collaborating with different restaurants and companies to ensure that female chefs actively participate in the business world.
- Female chefs can be encouraged to be role models for other women in the workplace and society, motivating them to pursue this profession and achieve a successful career.

The culinary profession has become a sector where women have successfully taken their place in recent years, but culinary is still perceived as a workplaces dominated by gender inequality. Therefore, supporting the careers of female chefs is of great importance. However, supporting the career development of female chefs should not be seen only as a situation that will empower women in the industry. It is believed that the industry, businesses, and employers can also provide certain advantages by supporting the career development of female chefs. Among these advantages, it is possible to express gains such as making the sector more varied and inclusive, increasing the creativity and collaboration of chefs with different perspectives and experiences, and being innovative and competitive. The economic empowerment of women, their role modeling, and leadership can lead to the emergence of gender equality in the workplace and society, contributing to positive developments for both workplace culture and society. Encouraging the performance, creativity, and leadership skills of female chefs in the workplace by the business can enhance productivity and profitability, contribute to the establishment of a strong and positive brand image, and increase corporate reputation.

## 8 Limitations and directions for the future research

The research is based on findings from participant statements regarding gender inequality in the culinary profession in the context of tourism. Research results are limited to the experiences, perceptions, and thoughts of the participants. In this context, future studies can focus on gender inequality in tourism-related services/jobs and comparative analyses can be carried out. Another limitation of the research is that it was conducted in a society with patriarchal traditions. Research can be conducted with the assumption that different results can be achieved by conducting the study in societies with matriarchal or feminine authority.

## Data availability statement

The datasets presented in this article are not readily available because the data collected is specific to the participants. Requests to access the datasets should be directed to tuba.gezen@atauni.edu.tr.

## Ethics statement

The studies involving humans were approved by Atatürk University, Social Sciences Institute Ethics Committee Number E.88656144–000-2300000159. The studies were conducted in accordance with the local legislation and institutional requirements. Written informed consent for participation was not required from the participants or the participants’ legal guardians/next of kin because An online and face-to-face survey was administered to university students.

## Author contributions

TT: Conceptualization, Data curation, Formal analysis, Investigation, Methodology, Project administration, Resources, Software, Validation, Supervision, Visualization, Writing – original draft, Writing – review & editing. NK: Conceptualization, Investigation, Resources, Writing – original draft.
